# Utero-vaginal aplasia (Mayer-Rokitansky-Küster-Hauser syndrome) associated with deletions in known DiGeorge or DiGeorge-like loci

**DOI:** 10.1186/1750-1172-6-9

**Published:** 2011-03-15

**Authors:** Karine Morcel, Tanguy Watrin, Laurent Pasquier, Lucie Rochard, Cédric Le Caignec, Christèle Dubourg, Philippe Loget, Bernard-Jean Paniel, Sylvie Odent, Véronique David, Isabelle Pellerin, Claude Bendavid, Daniel Guerrier

**Affiliations:** 1 CNRS UMR 6061, Institut de Génétique et Développement de Rennes, IFR 140 GFAS, Faculté de Médecine, 2 avenue du Professeur Léon Bernard CS 34317, 35043 Rennes Cedex, France; 2 Pôle d'Obstétrique Gynécologie et Médecine de la Reproduction, Hôpital Sud, 16 bd de Bulgarie BP 90437, 35203 Rennes Cedex, France; 3 Service de Génétique Médicale, Hôpital Sud, 16 bd de Bulgarie BP 90437, 35203 Rennes Cedex, France; 4 Service de Génétique Médicale, CHU de Nantes, 38 bd Jean Monnet, 44093 Nantes Cedex 1, France; 5 INSERM UMR 915 - Institut du Thorax, 8 quai Moncousu BP 70721, 44007 Nantes Cedex 1, France; 6 Laboratoire de Génétique Moléculaire et Hormonologie, CHU Pontchaillou, 2 rue Henri Le Guilloux, 35033 Rennes Cedex 9, France; 7 Service de Pathologie, Hôpital Sud, 16 bd de Bulgarie BP 90437, 35203 Rennes Cedex, France; 8 Service de Gynécologie-Obstétrique, Centre Hospitalier Intercommunal, 40 avenue de Verdun, 94000 Créteil, France

## Abstract

**Background:**

Mayer-Rokitansky-Küster-Hauser (MRKH) syndrome is characterized by congenital aplasia of the uterus and the upper part of the vagina in women showing normal development of secondary sexual characteristics and a normal 46, XX karyotype. The uterovaginal aplasia is either isolated (type I) or more frequently associated with other malformations (type II or Müllerian Renal Cervico-thoracic Somite (MURCS) association), some of which belong to the malformation spectrum of DiGeorge phenotype (DGS). Its etiology remains poorly understood. Thus the phenotypic manifestations of MRKH and DGS overlap suggesting a possible genetic link. This would potentially have clinical consequences.

**Methods:**

We searched DiGeorge critical chromosomal regions for chromosomal anomalies in a cohort of 57 subjects with uterovaginal aplasia (55 women and 2 aborted fetuses). For this candidate locus approach, we used a multiplex ligation-dependent probe amplification (MLPA) assay based on a kit designed for investigation of the chromosomal regions known to be involved in DGS.

The deletions detected were validated by Duplex PCR/liquid chromatography (DP/LC) and/or array-CGH analysis.

**Results:**

We found deletions in four probands within the four chromosomal loci 4q34-qter, 8p23.1, 10p14 and 22q11.2 implicated in almost all cases of DGS syndrome.

**Conclusion:**

Uterovaginal aplasia appears to be an additional feature of the broad spectrum of the DGS phenotype. The DiGeorge critical chromosomal regions may be candidate loci for a subset of MRKH syndrome (MURCS association) individuals. However, the genes mapping at the sites of these deletions involved in uterovaginal anomalies remain to be determined. These findings have consequences for clinical investigations, the care of patients and their relatives, and genetic counseling.

## Background

Congenital aplasia of the uterus and the upper two thirds of the vagina is diagnosed as Mayer-Rokitansky-Küster-Hauser (MRKH) syndrome in 90% of affected women presenting with primary amenorrhea and otherwise normal secondary sexual characteristics, normal ovaries and a normal karyotype (46, XX) [1]. The incidence of MRKH syndrome has been estimated to be 1 in 4500 female births [2-4]. The uterovaginal aplasia can be isolated (type I; OMIM 277000) but it is more frequently associated with other malformations (type II; OMIM 6601076). Type II is also referred to as the MURCS (Müllerian Renal Cervico-thoracic Somite anomalies) association. The most common associated malformations involve the upper urinary tract affecting about 40% of patients [5] and the cervicothoracic spine affecting about 30 to 40% of patients [5-7]. Renal malformations include unilateral agenesis, ectopia of one or both kidneys, horseshoe kidney, hydronephrosis [7], and even bilateral renal agenesis (Potter sequence) [8]. Rachidial malformations most commonly encountered are scoliosis, isolated vertebral anomalies (asymmetric, fused or wedged vertebrae), Klippel-Feil association or Sprengel deformity [7,9]. Less frequent associated anomalies include hearing defects in about 10 to 25% of patients [10,11]. Cardiac malformations, such as tetralogy of Fallot [12], atrial septal defect [13] or pulmonary valvular stenosis [14] are found in rare cases, as rare facial asymmetry [15-17] and digital anomalies, such as brachymesophalangy, ectrodactyly or duplicated thumb [18-20]. Type II MRKH or the MURCS association may be attributed to alterations in the blastema giving rise to the cervicothoracic somites and the pronephric ducts, the ultimate spatial relationships of which are already determined by the end of the fourth week of fetal development [21].

MRKH syndrome was initially considered to be sporadic. The involvement of non-genetic or environmental factors was suggested but rejected. The description of an increasing and significant number of familial cases confirmed the involvement of a genetic component. The syndrome appears to be transmitted as an autosomal dominant trait with incomplete penetrance and variable expressivity [2,22-24]. Observations are consistent with a polygenic or multifactorial cause involving either mutations in one or several major developmental genes or limited chromosomal imbalances. However, the etiology of MRKH syndrome remains poorly understood (see [1] for review). At the present time, only a SHOX duplication has been described associated with type I MRKH syndrome in some cases [25].

In respect to type II MRKH or MURCS association, the lack of clear genetic or chromosomal evidence led us to consider the wider spectrum of uterovaginal aplasia-associated malformations as a starting point for genetic investigations. We compared MRKH syndrome with other syndromes displaying phenotypic features overlapping with those of MRKH syndrome. Several of the anomalies found in the MRKH syndrome are also within the clinical spectrum of the 22q11.2 deletion syndrome, also referred to as DiGeorge syndrome (DGS syndrome; OMIM 188400) and velocardiofacial syndrome (VCF syndrome; OMIM 192430). Indeed, this phenotype involves congenital heart defects, such as tetralogy of Fallot, interrupted aortic arch, ventricular septal defect or persistent troncus arteriosus, dysmorphic facial features, cleft palate, hearing loss, thymic hypoplasia, hypoparathyroïdism, and developmental and behavioral problems [26-28]. Other less common manifestations include renal (horseshoe, hydronephrosis), vertebral (butterfly vertebrae, hemivertebrae, abnormalities of the cervical spine, scoliosis, Sprengel deformity) and extremity anomalies (polydactyly, syndactyly, club-foot) [28-30]. Ninety% of cases of DGS and 70% of cases of VCF syndrome are caused by a 1.5 to 3.0 Mb hemizygous deletion of chromosome 22q11.2 [31]. More precisely, the strictly DiGeorge syndrome appears to be associated with only *TBX1 *deletion or mutations [32]. However, DGS-like phenotypes have also been reported in patients with deletions of chromosome 4q34.2-qter [33,34], 8p23-pter [34-36], 10p14-p15 [34,37-41], 17p13 [42] or 18q21 [43], chromosomes 4, 8 and 10 being the most frequently chromosomes described associated with DGS-like phenotype. Some studies have described MRKH syndrome features associated with 22q11.2 deletions [8,44-47] and we showed a 4q34-qter deletion in one case of MRKH syndrome [48].

Here, we report a search in a cohort of 57 patients affected by the MRKH syndrome for deletions in the chromosomal regions most frequently associated with the DGS or the DGS-like phenotypes.

## Patients and methods

### Patients

We studied 55 women who presented utero-vaginal aplasia diagnosed by clinical examination and transabdominal ultrasonography and/or magnetic resonance imaging (MRI) or celioscopy. All patients had a normal 46, XX karyotype. The patients underwent a check-up to search for associated malformations including renal ultrasonography, spine radiography and echocardiography or audiogram, if required. Twenty (36.4%) presented isolated uterovaginal aplasia (MRKH type I). The other women (63.6%) variously displayed kidney defects, vertebral and other skeletal malformations (including Klippel-Feil and Sprengel anomalies, digital anomalies such as clinodactyly, brachydactyly and syndactyly), cardiac anomalies and hearing impairment. Two aborted fetuses with various abnormalities including uterovaginal aplasia were also analyzed.

All the subjects were enrolled through a French national multicentric research program, called PRAM (Programme de Recherche sur les Aplasies Müllériennes), which is registered in the Orphanet database (http://orphanet.infobiogen.fr). This study was approved by the local institutional review board, the "Comité de Protection des Personnes" (Project # 05/16-543), and is registered with the French Ministry of Health (DGS # 2005/030). Here, we only report the clinical features of the four subjects in whom we detected a deletion.

#### Case 1

This patient presented with primary amenorrhea, leading to a diagnosis of congenital absence of the upper vagina and uterus, with normal bilateral adnexa, as confirmed by celioscopy. Thelarche and pubarche were normal. The patient had no visceral malformations (the heart and kidneys, in particular, were normal, as assessed by ultrasound examinations) or hearing impairment. No skeletal abnormalities were observed. This patient was about 165 cm tall. Her schooling was standard and she was the only daughter of non consanguineous parents who suffered one miscarriage. Her father had no relevant medical background. Her mother was 152 cm tall and was 58 years old at the time of the study. At birth, the patient's mother had bilateral club feet. An atrial septal defect (ostium secundum type) was subsequently detected, requiring surgical correction at the age of 23. Bilateral serous carcinoma of the Fallopian tubes was diagnosed four years ago. The patient's mother had no renal defects or skeletal abnormalities. No mental impairment or other visceral malformations were reported in this family.

#### Case 2

This 19-year-old female patient was referred for the evaluation of primary amenorrhea. Thelarche and adrenarche had occurred at 13 years of age. Congenital uterovaginal aplasia with symmetric muscular buds, bilaterally normal ovaries and Fallopian tubes were demonstrated by celioscopy. Screening for anomalies commonly associated with MRKH syndrome revealed unilateral kidney agenesis but no other defects. In particular, this patient displayed no deafness or skeletal, heart, limb and facial malformations. She also had no psychiatric disorders. This patient had two sisters and two brothers, none of whom have any malformations. Her parents were phenotypically normal but were unavailable for further investigation. There was no family history of recurrent abortion or consanguinity.

#### Case 3

Case 3 was a fetus from a medically terminated pregnancy at 18 weeks of gestation following the ultrasonographic discovery of a bilateral renal agenesis. Fetopathological examination confirmed the absence of kidneys associated with uterovaginal aplasia. No other skeletal or heart malformation was observed. We later established that the father and the two sisters also displayed unilateral renal agenesis, without associated heart or skeletal malformation. Fetal ultrasound of the two sisters did not reveal any uterine malformation but no genital examination was performed (the children were too young). Case 3 had a normal 46, XX karyotype. The mother had no relevant medical history. One of the first-degree female cousins of the father presented unilateral renal agenesis also associated with ipsilateral half-uterus.

#### Case 4

This case was a female fetus obtained following medically termination of a pregnancy at 23.5 weeks of gestation due to bilateral renal agenesis. Fetopathological examination showed, in addition to the bilateral renal agenesis, uterovaginal aplasia, type B interrupted aortic arch, and thymic hypoplasia. Neither parent had morphological anomalies. This case has been preliminarily reported in a study of 49 fetuses with three or more significant anomalies of unknown etiology, in which a battery of CGH methods were applied to detect chromosomal imbalances [49].

### Healthy control subjects

About 15% of genes in the OMIM morbid map overlap CNVs (Redon, 2006). CNVs can cause Mendelian or sporadic traits, or be associated with complex diseases. Probands can inherit a disease-associated rearrangement from unaffected parents, which underscores the variable penetrance of some diseases resulting from dosage effects (Redon, 2006). However, CNVs can also be benign polymorphism variants. To confirm that the deletions observed in the present cases were not non-pathogenic genomic copy number variations (CNV), a cohort of 100 healthy normal subjects (50 men and 50 women) was tested. All volunteers were informed about the study and signed a consent form approved by the local Ethical Committee.

### Methods

Genomic DNA was extracted from whole blood of patients or from fetal tissues, whole blood of parents and healthy control subjects using the QIAamp DNA Kit (http://www.qiagen.com) according to the manufacturer's protocol.

#### MLPA

Multiplex ligation-dependent probe amplification (MLPA) was performed with samples from our cohort of 57 MRKH syndrome patients, the parents if possible and 100 healthy control subjects. Two independent experiments were carried out each on 100ng of genomic DNA. We used the SALSA MLPA kit P023-DiGeorge (MRC-Holland, Amsterdam, Netherlands) according to the manufacturer's instructions. This kit allows semi-quantitative analysis of 39 genomic sequences located on 4q (six targets), 7p15 (one target), 8p (five targets), 10p (five targets), 17p13 (four targets), 18q21 (two targets), 22q11 (ten targets) and 22q13 (one target); most of these loci are involved in DGS or DGS-like phenotypes. Amplification products were analyzed by capillary electrophoresis using an ABI 3100 Genetic Analyzer (Applied Biosystems). ABI result files were normalized using the GeneMarker Software (Softgenetics) and an in-house Excel spreadsheet. The results are represented as histograms.

#### Duplex PCR/liquid chromatography (DP/LC)

The chromosomal breakpoints in cases 2 and 3 were confirmed and mapped more precisely using multiplex PCR/liquid chromatography in a duplex assay according to a standard protocol [50]. Briefly, duplex PCR was used, associating unlabeled primers for an endogenous control gene, *HMBS*, and for the region showing imbalance in the tested patient. This enables simultaneous amplification of the two targets under semi-quantitative conditions. Primers were designed using Primer Premier Software (Premier Biosoft International, Palo Alto, CA, USA). Navigator™ Software (Transgenomic, Omaha, NE, USA) was used for data analysis and the *HMBS *peak was used for normalization; relative peak intensities for each amplicon directly reflected genomic copy number.

#### Array-CGH

Oligonucleotide array-CGH analysis was performed using the Agilent Human Genome CGH microarray 44 K (Agilent Technologies, Santa Clara, CA, USA) as described in version 4.0 of the protocol provided by Agilent (Agilent Oligonucleotide Array-Based CGH for Genomic DNA Analysis). This platform is constituted of 44,290 60-mer oligonucleotide probes for mapped genes or unique DNA sequences with an average spatial resolution of 35 Kb. Patient and same gender reference genomic DNA (gDNA) were digested, labeled with Cy5™-dUTP and Cy3™-dUTP using the Agilent Genomic DNA Labeling Kit PLUS and cohybridized to Agilent 4 × 44 k arrays. After washes, hybridized slides were scanned on the Agilent scanner G2565BA. Images were analyzed with Agilent Feature Extraction Software version 9.1 (CGH-v4_91 protocol). Data were imported into Agilent CGH analytics software version 3.4.27 (statistical algorithm: z-score, sensitivity threshold: 2.5, moving average window: 5 points) for a graphical overview and analysis.

## Results

**Figure 1:**
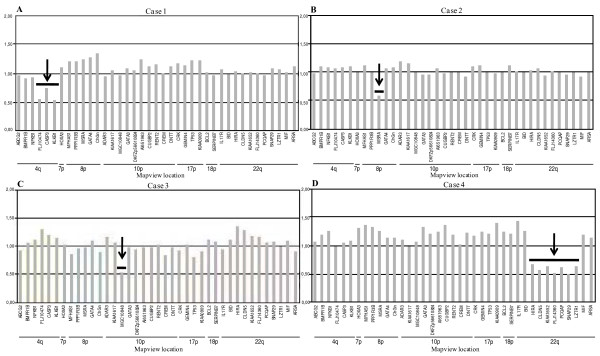
**MLPA analysis of cases 1 to 4**. Gene dosage in cases 1 to 4 assessed by the MLPA P023 kit. Histogramms represent allelic dosage of each target gene. Black arrows show deletion of (A) FLJ10474 (*ODZ3*), CASP3 and KLKB1 probes at 4q34-qter in case 1; (B) MSRA probe at 8p23 in case 2; (C) MGC10848 (*ITIH5*) probe at 10p14 in case 3; (D) HIRA, CLDN5, KIAA1652 (*TXNRD2*), FLJ14360 (*KLHL22*), PCQAP, SNAP29, LZTR1 probes at 22q11.2 in case 4.

The 57 patients and 100 healthy control subjects were tested by MLPA with the P023 kit. None of the control subjects showed variation in the copy number for any of the markers used in this kit. The analysis disclosed a deletion at different chromosomal regions in each of four patients (Figure 1): deletion of three probes (FLJ10474 corresponding to *ODZ3 *gene, CASP3, KLKB1) at 4q34-qter in case 1; one probe (*MSRA *gene) at 8p23 in case 2; one probe (MGC10848 corresponding to *ITIH5 *gene) at 10p14 in case 3; and seven probes (KIAA1652 and FLJ14360 corresponding to *TXNRD2 *and *KLHL22 *genes, respectively, HIRA, CLDN5, PCQAP, SNAP29, LZTR1) at 22q11.2 in case 4.

**Figure 2:**
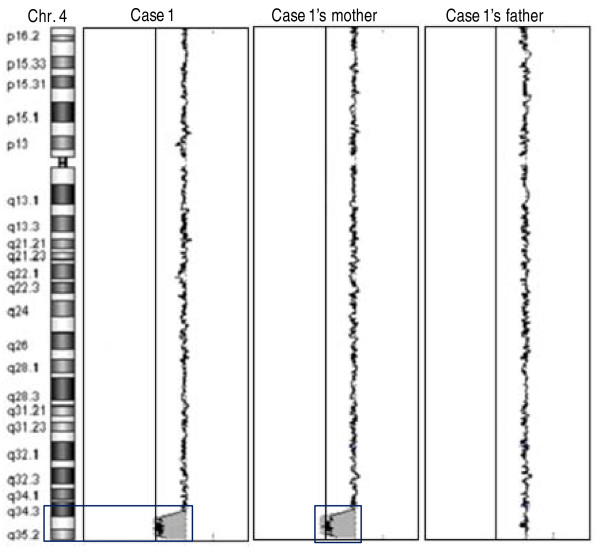
**Array-CGH profile of chromosome 4 in case 1 and her parents**. A deletion of about 8 Mb was detected in 4q34-qter in case 1 and her mother (black boxes). No deletion was found in her father. Log2 ratio values for all probes are plotted as a function of their chromosomal position. Solid and dotted lines indicate the log2 ratio thresholds -0.25 (loss) and 0.25 (gain), respectively.

For case 1, testing with the MLPA P023 kit revealed an identical deletion of about 8 Mb at 4q34-qter in the mother, but no deletion was found in the father. A complementary DP/LC analysis, reported previously, corroborated these results [48]. We confirmed and refined this former analysis by array-CGH and demonstrated that there were no other chromosomal abnormalities in either the proband or her mother, and no abnormality in her father (Figure 2).

**Figure 3:**
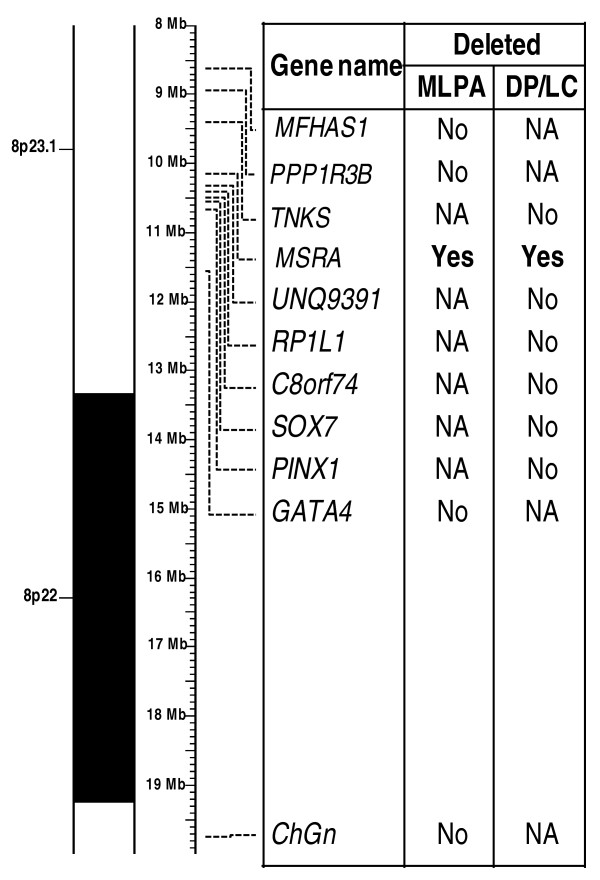
**Details of the 8p23 deletion found in case 2**. Summary of gene quantification and chromosome breakpoint refinement: several genes were tested by MLPA (P023 kit) and DP/LC. The combined results show an about 1.2 Mb deletion delimited by *TNKS *and *UNQ9391 *gene markers and including *MSRA*. NA: Not Applicable.

MLPA analysis of case 2 led to detect a single copy of the *MSRA *gene on chromosome 8p23 but two copies of the flanking genes, *PPP1R3B *and *GATA4*, were found in the same MLPA experiment, leading to estimate the maximum size of the deletion to be 2.6 Mb. The chromosomal breakpoint was more accurately mapped using DL/PC with amplicons located in *TNKS, MSRA *(used to corroborate MLPA results)*, UNQ9391, RP1L1, SOX7, PINX *genes and in the *C8orf74 *open reading frame: the maximum size of the deletion was then found to be 1.2 Mb in between *TNKS *and *UNQ9391 *(Figure 3). However, we could not investigate either this case by array-CGH (insufficient genomic DNA was available) or her parents because we were unable to contact the family again.

**Figure 4:**
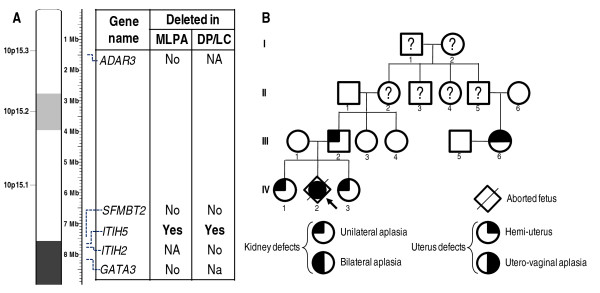
**Details of the 10p14 deletion in case 3**. **A. **Summary of gene quantification and chromosome breakpoint refinement: several genes were tested by MLPA (P023 kit) and DP/LC. Combination of the results indicates a heterozygous deletion of about 230 Kb including the *ITIH5 *gene. NA: Not Applicable. **B. **Pedigree of the case 3's family. The proband is indicated with an arrow. Question marks indicate that the phenotype of putative genetic carriers is unknown.

MLPA analysis of case 3 revealed a deletion of 0.9 Mb maximum in the 10p14 region which includes the whole *ITIH5 *gene. DP/LC of the *SFMBT2 *and *ITIH2 *genes flanking the *ITIH5 *gene mapped this deletion to a maximal size of 230 Kb, including only the *ITIH5 *gene (Figure 4A). In addition, array-CGH analysis showed no deletion of the markers flanking the *ITIH5 *gene or in other parts of the genome (data not shown). Thus, this deletion appears to be very small. MLPA, DP/LC and array-CGH analysis identified no deletion in the father, the two sisters, the first-degree paternal female cousin who presented unilateral renal agenesis, or in the phenotypically normal mother and two paternal aunts. The pedigree of this family is shown in Figure 4B.

MLPA analysis of case 4 confirmed and refined the preliminary results published on this case. Deletion of three clustered probes (RP1-157E19, RP1-238C15, and RP11-316L10) at 22q11.2 was found by array-CGH and confirmed by QMPSF (Quantitative Multiplex PCR of Short Fluorescent fragments) (TBX1 amplicon) [49]. Comparison of the 6.3 Mb deletion initially found by array-CGH and that of 5.95 Mb we detected by MLPA indicated that the deletion was 5.24 Mb maximum.

## Discussion

We report four chromosomal deletions located at 4q34-qter, 8p23, 10p14 and 22q11.2, all known to be associated with DGS or DGS-like phenotypes, in four independent MRKH subjects.

To differentiate clinically relevant deletions from non pathogenic chromosomal variants, we first consulted the Database of Genomic Variants (http://projects.tcag.ca/variation/) to exclude copy number variations (CNVs) previously observed in control healthy individuals. CNVs within each of the loci investigated in our study were reported to be absent, smaller or very rare (<0.5%). To distinguish between potentially pathogenic variants and unidentified benign CNVs, we tested 100 DNA samples from healthy volunteers with the same MLPA kit. No variation in copy number was observed in these controls for any of the markers used in this kit. The size of the deletions found in the described cases and the absence of deletion at these loci from all controls, strongly suggest that the four deletions we identified are pathogenic.

Deletion of 4q34-qter may lead to a phenotype similar to the DiGeorge phenotype [33,34], including congenital heart disease (atrial septal defect, ventricular septal defect), cleft palate, and learning difficulties. Few cases of 4q34-qter deletion have been reported [33,51,52] and there are only three reports of familial transmission affecting the mother and her sons showing features of DiGeorge syndrome [52-54]. The present case 1 is the fourth described case of familial transmission of 4q34-qter deletion and the first case associated with MRKH syndrome inherited from a mother displaying different clinical features. Several genes are included in this deletion: the *FAT tumor suppressor 1 *(*FAT1*) gene, which belongs to the cadherin superfamily [55], appears to be a common candidate gene for malformations observed in both the daughter and the mother [48]. First, *FAT1 *has been described as one of the five tumor suppressor gene located on the long arm of chromosome 4 [56-59] and was evidenced as an anti-proliferative factor of smooth muscle cells [60]. So, it might account for the bilateral serous carcinoma of the Fallopian tubes, discovered in case 1's mother when she was 54, as previously discussed [48]. Second, the *FAT1 *gene appears to be also a putative candidate gene for a developmental failure of Müllerian differentiation in the embryo. Indeed, the protein encoded by this gene has been shown to take an important part to developmental processes requiring cell polarization [61], cell-cell interactions [55] and epithelium-mesenchyme interaction [62] such as tubulogenesis [63]. In particular, it seems to be involved in smooth muscle differentiation process [62] and could therefore give an explanation to the Müllerian duct differentiation arrest observed in MRKH syndrome. In this hypothesis, haploinsufficiency of the *FAT1 *gene would lead to unequal consequences, probably depending on each patient's genomic background and affecting embryonic or adult tissues. It may therefore be helpful for future studies to request further information about internal genital anomalies and cancer susceptibility in individuals with 4q deletions.

Small interstitial deletions within the 8p23.1 region, such as that found in case 2, have been associated with severe congenital heart disease, mental retardation, microcephaly, and a characteristic behavioral phenotype, all these features being included in DGS-like phenotype [35,36]. The *GATA4 *gene at 8p23.1-pter has been implicated in all such cases with a heart defect [36,64,65] but not in patients without heart defects [36]. The region deleted from our patient mapped close to *GATA4*, but did not include this gene, consistent with the phenotype of this patient. Deletion of 8p23.1 has also been shown to be associated with renal anomalies (hydronephrosis, horseshoe kidney), vertebral anomalies, and polydactyly [64-67]. All these features are included in the associated malformations of type II MRKH (MURCS association) [1]. This suggests that the different clinical features observed in case 2 may be caused by the deletion of clustered genes within the commonly deleted region and that the 8p23.1 chromosomal region corresponding to a DGS-like morbid locus includes at least one gene involved in MRKH syndrome. This region contains the peptide methionine sulfoxide reductase (*MSRA*) gene [68], the retina-specific *RP1L1 *gene [69], MIR 124-1, a micro-RNA preferentially expressed in the developing brain [70], the *TNKS *gene involved in regulating telomere length [71], and other genes of unknown function. None of the known genes in this region is particularly likely to be involved in MRKH syndrome. Finding redundant deletions in a larger cohort of patients would certainly be of great help to map the MRKH-associated genomic region on 8p23.1 and identify candidate gene(s).

Chromosome 10p terminal deletions have also been associated with DiGeorge-like phenotypes [39,40,72]. Studies of patients with 10p deletions have allowed the definition of two non-overlapping regions that contribute to this complex phenotype: the DiGeorge critical region 2 (DGCR2) [39,72] located on 10p13-14 associated with heart defects and T-cell deficiency; and the HDR region located on 10p14-10pter associated with *h*ypoparathyroïdism, sensorineural *d*eafness, and *r*enal defect [73]. This implies that the DGS-like phenotype associated with 10p deletion can be considered as a contiguous gene syndrome [74]. *GATA3 *haploinsufficiency is the underlying defect in the HDR syndrome [73] and *BRUNOL3 *is a candidate gene for thymus hypoplasia and possibly for heart defects [75]. Our case 3 is the first reported case of 10p14 deletion associated with uterovaginal aplasia. This deletion seems to affect only the *ITIH5 *gene, located in the HDR locus, distal to the *GATA3 *gene and might account for genital abnormalities associated with renal defects. This suggests that the *ITIH5 *gene may be involved in a common mechanism of renal and genital tract differentiation and that *GATA3 *haploinsufficiency independently only causes renal defects.

**Figure 5:**
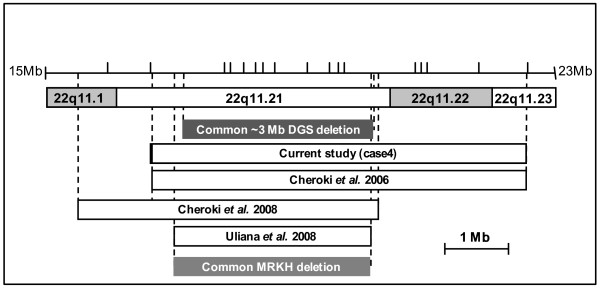
**Diagram of 22q11.1-q11.23 chromosomal region**. Schematic representation of the 22q11.1-q11.23 chromosomal region between 15 and 23 Mb from the telomeric end of the short arm. Dark gray box represents the ~3Mb most common 22q11 deletion associated with DGS syndrome; white boxes represent the maximum distance between undeleted markers in this current study (case 4) and the previously reported MRKH cases. Light gray box shows the smallest 22q11.2 deletion common to known MRKH syndrome patients.

Finally, the 22q11.2 deletion is the genetic etiology of about 90% of cases of DiGeorge syndrome [31]. Six cases of uterovaginal aplasia have been reported with 22q11.2 deletion. In 1997, Devriendt *et al. *described a 19-week female fetus with MRKH syndrome, unilateral renal agenesis, and contralateral multicystic renal dysplasia [8]. In 2006, Cheroki *et al. *analyzed five cases of uterovaginal aplasia associated with other malformations by array-CGH: a 22q11.2 deletion (~4 Mb in size) was detected in a young woman presenting tract genital, heart, skeletal and facial anomalies but no renal defect [44]. In the four other cases, uterovaginal aplasia was associated with unilateral renal agenesis and other manifestations (facial anomalies, mild developmental delay, hypoparathyroïdism, skeletal or heart defect) [45-47]. Here, we report an additional case (case 4) of 22q11.2 deletion associated with uterovaginal aplasia, thymic hypoplasia, interrupted aortic arch type B, and for the first time, bilateral renal agenesis. The smallest common deleted region among the deletions overlapping 22q11.2 and associated with MRKH type II (MURCS association) is the most frequent ~3 Mb 22q11.2 deletion associated with DGS [76] (Figure 5). This strongly suggests that the MURCS association is an additional component of the 22q11.2 deletion phenotype. However, the genes within 22q11.2 deletion involved in both renal and uterovaginal anomalies remain to be determined.

## Conclusion

The MRKH syndrome is characterized by congenital uterovaginal aplasia frequently associated with extragenital anomalies (MURCS association). These other manifestations, such as renal, skeletal and heart malformations are also found in DGS or DGS-like phenotypes. Our results show that uterovaginal aplasia can also be associated with deletions in known DGS (22q11.2) or DGS-like (4q34-qter, 8p23, and 10p14) loci. These data suggest that the MURCS association may be an additional feature of the broad phenotypic spectrum of DiGeorge syndrome. In the light of this information, the extent of malformations in cases of either MRKH syndrome or DGS syndrome should be reconsidered. Patients with MRKH syndrome, especially MURCS association, should undergo evaluation of chromosomal regions responsible of DGS or DGS-like phenotypes. Similarly, patients diagnosed for DGS syndrome should be assessed for genital malformations.

## Competing interests

The authors declare that they have no competing interests.

## Authors' contributions

KM, TW, LR and DG carried out the molecular genetic studies. CLC performed preliminary studies on case 4 and provided full clinical description and genomic DNA samples. CD and VD supervised most of the genomic DNA samples preparations, quality control and follow-up, and participated in the design of the study. PL carried out fetal examination of case 3 and provided us with tissue samples. BJP diagnosed some patients of the cohort, provided us their blood samples and was of great help in the initial setting of the PRAM clinical network. LP and SO were involved in the diagnosis of some patients and in the recruitment of some volunteers. IP and DG initiated the overall research program, CB participated to the design of the present study, KM and DG wrote the manuscript, DG mainly conceived of the study, and participated on its design and coordination. All authors read and approved the final manuscript. The authors declare that they have no competing interests.
